# VH1 Family Immunoglobulin Repertoire Sequencing after Allogeneic Hematopoietic Stem Cell Transplantation

**DOI:** 10.1371/journal.pone.0168096

**Published:** 2017-01-17

**Authors:** Maya K. Sethi, Felicitas Thol, Michael Stadler, Michael Heuser, Arnold Ganser, Christian Koenecke, Oliver Pabst

**Affiliations:** 1 Institute of Immunology, Hannover Medical School, Hannover, Germany; 2 German Centre for Infection Research (DZIF), partner site Hannover-Braunschweig, Hannover, Germany; 3 Department of Hematology, Hemostasis, Oncology and Stem Cell Transplantation, Hannover Medical School, Hannover, Germany; 4 Institute of Molecular Medicine, RWTH Aachen, Aachen, Germany; Beth Israel Deaconess Medical Center, UNITED STATES

## Abstract

After allogeneic hematopoietic stem cell transplantation (HSCT), recovery of humoral immunity is essential to protect from life-threatening infections. However, monitoring the humoral immune system after transplantation with standard techniques in the clinical routine is imprecise. Here, we performed sequencing of mononuclear bone marrow cells to characterize the VH1-repertoire of switched B cells of healthy volunteers and patients undergoing HSCT. Analysis of healthy bone marrow donors and patients showed virtually no clonally related sequences between individuals. Interestingly, clonally related sequences were present in pre- and post-transplantation bone marrow of patients undergoing HSCT for acute myeloid leukemia treatment. We consistently observed such related B cell clones, irrespective of conditioning regimen, donor source or time post transplantation. In general, repertoire diversity was lower in post-HSCT as compared to pre-HSCT samples. However, post-HSCT repertoires retained highly mutated sequences, despite immunosuppressive therapy and presence of T cell deficiency after HSCT. These observations identify key properties of the recovering B cell compartment and provide a conceptual framework for the surveillance of humoral immunity after allogeneic transplantation.

## Introduction

Allogeneic hematopoietic stem cell transplantation (HSCT) is a successful therapeutic option for a number of malignant and benign diseases of the lympho-hematopoietic system. HSCT patients undergo conditioning therapies to treat the underlying disease and to eradicate the recipient’s hematopoietic system. Immune cell recovery is generally thought to be donor-derived, but especially after reduced intensity conditioning (RIC) host immune cells can persist in a state of mixed hematopoietic chimerism [1]. E.g. plasma cells seem to be particularly resistant to conditioning procedures and recipient plasma cells were shown to persist in the bone marrow after transplantation [2, 3]. Indeed, in some patients, recipient type immunoglobulins against antigens encountered before transplantation can be detected for years after HSCT [4–6].

Whereas recovery of many innate immune cell types occurs within several weeks, reconstitution of the immune lymphocyte system takes several months to years (reviewed in [7]). In fact, the first B cells detected early after HSCT in the peripheral blood are usually of donor origin and full restoration of a donor derived B cell compartment is thought to take up to two years in patients without additional immunosuppressive therapy or graft-versus-host disease (GvHD) [8, 9]. Thus, HSCT goes along with a prolonged immunodeficiency in particular of the adaptive immune system, increased risk of infection and deregulated B cell responses as seem in patients with ‘monoclonal gammopathy of undetermined significance’ (MGUS) [10].

Dynamics of the lymphocyte compartment after HSCT have been estimated by spectratyping, which determines the length distribution of the complementary determining region 3 (CDR3) and analysis of gene segment usage. Spectratyping of the B cell compartment indicated a diverse B cell repertoire 90 days after HSCT [11], whereas variable gene segment usage suggested slow recovery of a diverse population of naïve B cells [12]. However, both methods do not allow defining individual B or T cell clones and thus are limited to a rough description of the lymphocyte repertoire. Therefore, in this study we explored feasibility of next generation sequencing (NGS) based B cell repertoire analysis to characterise the composition and reconstitution of the B cell compartment in HSCT patients. NGS can provide in depth information with respect to lymphocyte repertoire diversity, clonal sizes and composition as well as mutation frequencies (reviewed in [13, 14]). We speculate that such information might help to estimate the state of immune reconstitution after HSCT.

Here we analyzed the switched VH1immunoglobulin (Ig) repertoire in patients with acute myeloid leukemia (AML). We provide evidence for the persistence of individual recipient B cell clones, irrespective of conditioning therapy and type of stem cell graft for at least two years post transplantation. Post-HSCT VH1 family repertoires showed low diversity but retained high numbers of somatic mutations, irrespective of the patient’s clinical course. These findings demonstrate feasibility of Ig repertoire sequencing as a powerful tool for the surveillance of humoral immunity after transplantation.

## Results

### Ig switch repertoires do not overlap between individuals

We have previously reported Ig repertoire sequencing to analyze the IgA repertoire in murine and human intestine [15, 16] and to compare clonal relatedness of Ig isotypes [17, 18]. Here we employed Ig repertoire sequencing to describe the composition of the Ig compartment in the bone marrow of patients undergoing HSCT for acute myeloid leukemia treatment.

**Fig 1:**
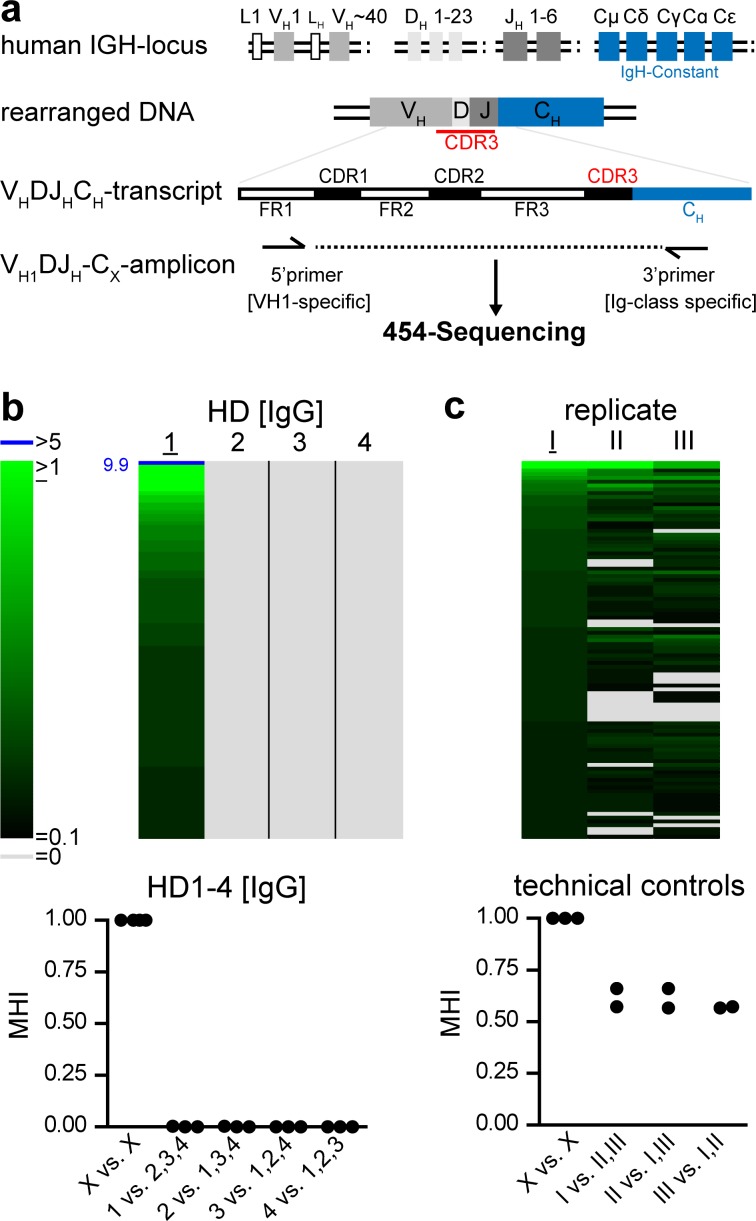
Human bone marrow samples show highly individual Ig-Repertoires. (a) Schematic overview of human VDJ-amplicons. Amplicon libraries for each Ig class contain framework (FR2, 3) and complementary determining regions (CDR1, 2, 3). (b) In a set of 4000 IgG-sequences per bone marrow sample, clones with 95% CDR3 sequence identity and identical VJ-usage were clustered as clonotypes. Frequencies of clonotypes are indicated by color code; clonotypes comprising more than 5% of the repertoire are highlighted in blue and numbers indicate their frequencies; grey color indicates absence of a given clonotype. Clontypes were sorted according to their frequency in healthy donor (HD) 1 and the 100 most frequent clonotypes of HD1 and their abundance in HD2-4 were displayed as heatmap. Similarity of repertoires was expressed as Morisita-Horn index (MHI), comparing 4000 clustered CDR3 sequences of HD1-4. Symbols represent pairwise comparisons. (c) The IgG-repertoire of HD4 (I) was re-investigated as independent V_H1_DJ-amplificate (II) or de-novo cDNA plus V_H1_DJ-amplificate (III). Repertoire-similarity of I to its technical replicates II and III is illustrated by heatmap and MHI, evaluating 2500 clustered sequences of each sample.

We first established basic properties of the immunoglobulin repertoire of switched B cell subsets (the switch Ig repertoire) in the bone marrow of four healthy donors. RNA was isolated from at least 10^7^ total bone marrow cells and transcribed into cDNA. The frequency of plasma cells in bone marrow typically is in the range of 1% [19], suggesting that each sample contained at least 100.000 plasma cells besides other Ig encoding B cell subsets. Variable heavy chain regions of the VH1 family were amplified by PCR using primers binding to the human VH1 domain and the constant region of either IgG, IgA or IgE encoding transcripts (Fig 1A), i.e. of each sample we generated three independent amplicon libraries representing the different isotypes. Sequences were obtained by Roche 454 NGS technology and compared against the IMGT® reference database (http://www.imgt.org/) [20, 21]. Only productive sequences were considered for further analysis thereby reducing the number of sequences carrying sequencing mistakes. Expectedly, more than 98% of sequences were assigned to the VH1 family. The VH1 family represents about 10% of all Ig sequences in humans, suggesting that even though we did not cover the whole Ig repertoire, VH1 family-restricted repertoire information can provide relevant insights into the B cell compartment. For further analysis, sequences that showed identical V and D usage and 95% sequence identity of their CDR3 were considered as clusters of clonally related sequences, i.e. contained sequences originating from a shared B cell clone [15].

To visualize the architecture of the switch Ig repertoire and to compare the presence/absence of clonally related sequences between samples, we displayed the frequency of each sequence cluster in a heat map [16–18]. In the heat map, sequence clusters were ranked from cluster comprising the most sequences to clusters containing fewer sequences. Thus each line represents one B cell clone/cluster and the color code reflects its frequency within the overall data set. Some sequence clusters were highly expanded and contributed more than 1% of all clusters (depicted in bright green or in blue color for clusters that contributed more than 5%) in the heat map whereas other clones were much less abundant (depicted as dark green in the heatmap) (Fig 1B and 1C). Virtually no clonally related sequences were observed when the Ig repertoire of different individuals was compared (Fig 1B), most likely reflecting the enormous diversity of CDR3 sequences generated during B cell development.

In contrast, when we compared the Ig repertoire information between replicate samples, clonally related sequences were abundantly shared between all sequence data sets (Fig 1C). Moreover, the frequency of expanded clones was highly correlated for replicates (S1 Fig), suggesting that PCR amplification did not result in detectable skewing of the sequence data and that sequence numbers obtained are indicative of true transcript levels present in the starting material.

To systematically quantify similarity/dissimilarity between Ig repertoires we used the Morisita-Horn similarity index (MHI). The MHI scores two identical populations as 1 whereas two completely non-overlapping populations are scored as 0 [15, 16]. The MHI takes into account both, the number of shared CDR3 sequences (clonal sharing) as well as the frequencies of shared clones. Consistent with the visual impression from the heatmap generated for the comparison of different bone marrow donors, MHI comparing switch Ig repertoire similarity between all four healthy donors were close to 0 (Fig 1B, mean±SD MHI observed for comparison between 4 healthy donors: 0.0004±0.0011). In contrast, MHI obtained for replicates samples, i.e. repertoire data sets generated from the identical patient material, were above 0.6, indicating strong repertoire similarity (Fig 1C).

These findings suggest that the Ig switch repertoire in each individual bone marrow is characterized by a unique selection of B cell clones. Thus, we speculate that the appearance of clonally related sequences in Ig repertoires generated for different isotypes or different starting material can only result from true presence of shared progenitors. We further explored the use of Ig repertoire sequencing to define the clonal relatedness of different Ig isotypes as well as to discriminate between donor and recipient derived Ig sequences in the bone marrow of HSCT patients.

**Fig 2:**
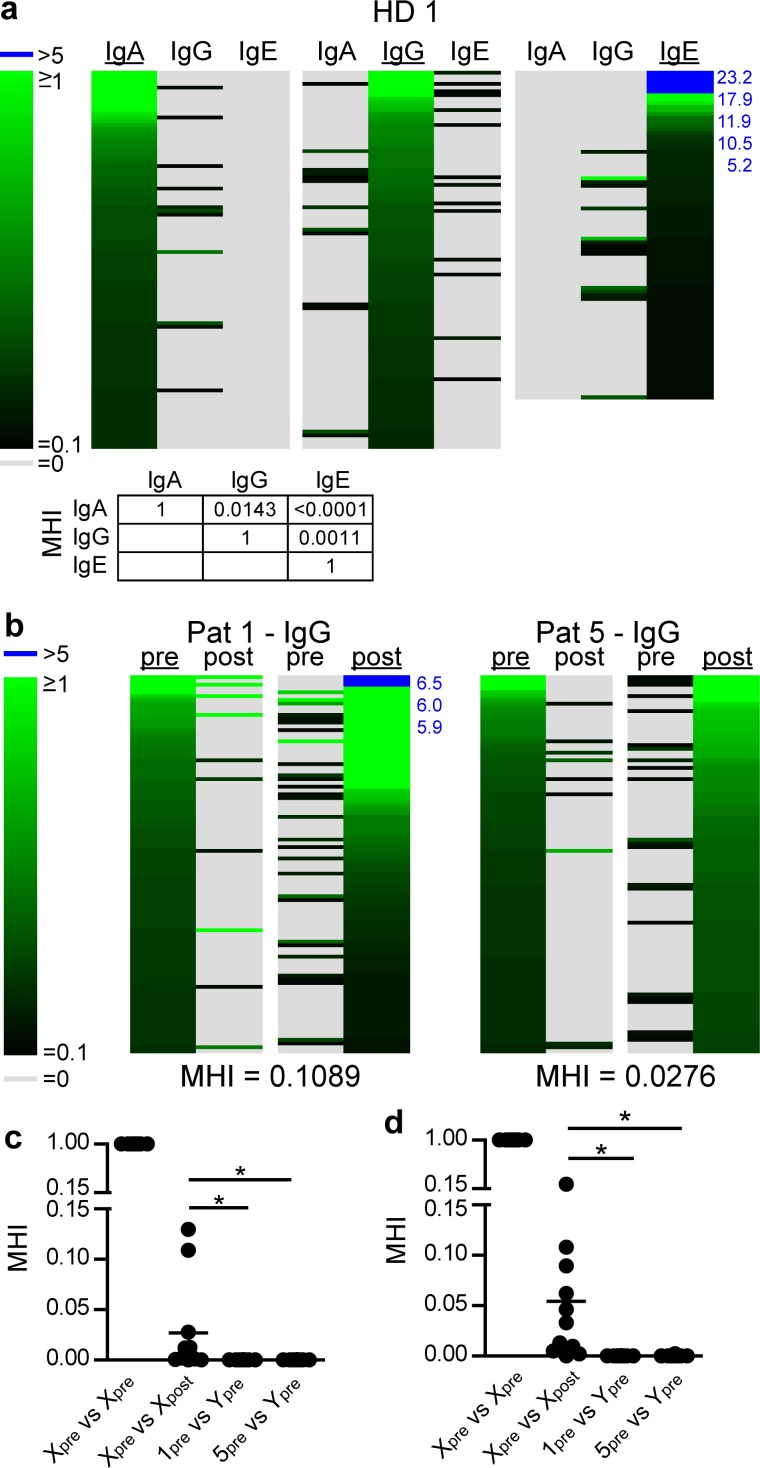
Ig repertoire analysis reveals clonal sharing between isotypes and persistence of clonotypes after HSCT. (a) IgA, IgG and IgE repertoires sequences were clustered (4000 sequences; 95% CDR3 sequence identity; same VJ-usage), and sorted according to the 100 most abundant clonotypes present in the IgA (left panel), IgG (middle panel), or IgE (right panel) repertoire and plotted as heatmap (see also Fig 1). Comparison of repertoire similarity of IgA, IgG and IgE repertoires of HD1 based on the analysis of 4000 sequences each. (b, c) IgG sequences of eleven patients pre- and post-HSCT (patient 1: 2000 sequences; other patients: 4000 sequences) were assigned to clonotypes. (b) Heatmaps show CDR3-overlap of the 100 most abundant IgG clonotypes of patients 1 and 5 pre- and post-HSCT. Repertoire similarity is described as Morisita-Horn index (MHI). (c) IgG-repertoire and (d) IgA-repertoire similarity quantified as MHI. Symbols represent comparisons of patient 1-11(X) pre- versus post-HSCT (X_pre_ vs. X_post_) or versus itself as control (X_pre_ vs. X_pre_). In addition, repertoires of patients 1 and 5 pre-HSCT were compared to all other patients pre HSCT (X_1_ or X_5_ vs.Y_pre_). Pairwise match of all pre-HSCT or post-HSCT IgG repertoires investigated results in a mean MHI of 0.0001±0.0005 for all pre- and 0.0001±0.0004 and for all post-HSCT samples (mean±SD). Pairwise match of all pre-HSCT or post-HSCT IgA repertoires investigated results in MHI of 0.00007±0.00033 for all pre- and 0.00002±0.00011 for all post-HSCT samples (mean±SD). Statistical analysis was performed using Kruskal-Wallis test with Dunn’s multiple comparison post hoc test, * p<0.05.

To quantify the degree of clonal sharing between isotypes, IgA, IgG and IgE repertoires within one bone marrow sample were compared. Indeed, we observed clonally related sequences among IgA and IgG and at lower degrees for IgG and IgE repertoires. Clonal sharing between IgA and IgG considering the 100 most frequent clones of each repertoire was in the range of 11 to 13%, i.e. more than 10% of the abundant clusters observed in the IgA repertoire were also detected in the IgG repertoire. MHI values based on 4000 sequences per repertoire by far exceeded values typically observed for the comparison of repertoire similarity between individuals (Fig 2A and S2A Fig). This indicates that different Ig isotypes are generated from clonally related B cells. Ig sequences contain characteristic sequences within their constant region that allow stratifying sequences into the distinct isotypes. In line with the literature, IgA sequences obtained from bone marrow samples were mostly of the IgA1 isotype and only about 10% of sequences were classified as IgA2 (S2B Fig). Stratification of IgG sequences showed highest proportions of IgG1, followed by IgG2, IgG3 and only low frequencies of IgG4 (S2B Fig). Quantification of repertoire similarities between isotypes showed that generally IgG sequences are more closely related to IgE compared to IgA sequences. Even higher similarities were observed comparing IgA1 to IgA2 and IgG1 to IgG2. In fact, evaluation of HD1-4 repertoires showed that among the 100 most frequent clonotypes, 21.75±9.81% (mean±SD) of clonotypes present in IgA1 repertoires were shared with IgA2 repertoires, and 24±12.55% (mean±SD) of IgG1 sequences were clonally related to IgG2 (S2C Fig). Collectively these results show that Ig sequencing of bone marrow cells allows describing repertoire similarity between samples. Ig repertoire comparison between individuals shows private repertoires that barely comprise clonally related sequences. In contrast, repertoire comparison within individuals reliably revealed clonotypes contributing to different Ig isotypes.

### Unique recipient derived Ig-clonotypes persist after allogeneic HSCT

Since Ig repertoires do not overlap between individuals, highly mutated immunoglobulin variable region sequences shared between the recipient’s bone marrow before and after HSCT most likely represent B cell clones that were resistant to the condition regimen or graft-versus-host reactions. Similarly comparison of the switch Ig repertoire in the donor bone marrow and the recipient bone marrow post HSCT may allow for the detection of potential donor derived B cells settling the recipient immune compartment. We obtained pre- and post-transplantation bone marrow samples of eleven AML patients (Table 1). Our cohort comprised patients treated with standard conditioning regimens (STD) as well as patients treated with RIC prior transplantation as well as all three standard stem cell grafts currently used for HSCT, peripheral blood stem cells (PBSC), bone marrow (BM) and umbilical cord blood (CB) transplantation. For further clinical information see also S1 Table. VH1 family Ig repertoire information was obtained for IgG and IgA.

**Table 1:** Clinical characteristics of AML-patients investigated for repertoire analysis before and after allogeneic HSCT.

Patient number	Age at Tx (years)	Sex	Graft	Conditioning regimen	Sampling pre HSCT (days before Tx)	Sampling post HSCT (days after Tx)	Drug-induced immunosuppression	Comments
1	49	m	MUD-PBSC	STD	268	37	yes	-
2	49	m	MUD-PBSC	STD	144	107	yes*	aGvHD °I^#^
3	20	f	MUD-PBSC	STD	70	139	yes**	-
4	41	m	MMUD-PSC	RIC	74	318	yes	poor graft function^§^/aGvHD °III^##^
5	57	m	MUD-PBSC	RIC	57	371	no	AML-Relapse
6	68	m	MUD-PBSC	RIC	449	251	no	MGUS/aGVHD °II^#^
7	40	m	MUD-PBSC	RIC	8	791	no	MGUS/AML-Relapse/PTLD^###^
8	32	m	MRD-BM	STD	207	21	yes	-
9	74	f	MUD-BM	RIC	9	42	yes	-
10	55	f	DCB	STD	720	87	yes	poor graft function^§^
11	64	f	DCB	RIC	567	180	no	poor graft function^§^

**Abbreviations:** aGVHD, acute graft-versus-host disease; AML, acute myeloid leukemia; BM, bone marrow; DCB, double cord blood; f, female; Ig, immunoglobulins; m, male; MGUS, monoclonal gammopathy of unknown significance; MMUD, mismatched unrelated donor; MRD, matched related donor; MUD, matched unrelated donor; PB, peripheral blood; PTLD, post-transplant lymphoproliferative disease; PBSC, peripheral blood stem cells; RIC, reduced intensity conditioning; STD, standard conditioning; Tx, transplantation

* (no, discontinued 1 week before).

** (no, discontinued 3 days before).

# (no systemic aGVHD treatment).

## (systemic aGVHD-therapy with prednisolone).

### (PTLD-therapy with prednisolone, Rituximab and Cyclophosphamide).

§ (poor graft function was defined as incomplete hematopoietic recovery without evidence of disease relapse).

Similar to our observation in healthy individuals, comparison of repertoire similarity between patients showed virtually no overlap, i.e. we did not detect clonally related sequences between patients (data not shown). In contrast, comparison of pre- and post-transplantation samples of the same patient showed considerable similarity (Fig 2B–2D and S3–S5 Figs). Seven out of eleven patients showed MHI at least 10 fold higher compared to the mean MHI observed for the comparison between individual patients. Thus, in these cases similarity of pre- and post-transplantation IgG repertoires clearly exceeded the very low frequency of shared clones observed for inter-individual sample comparisons. Even higher similarities were observed for pre- and post-transplantation IgA repertoires. All patients, except one, showed clonally related sequences in the pre- and post-transplantation samples, most likely representing IgA-switched B cells of recipient origin that persisted after HSCT.

Pre and post transplantation repertoire similarities showed no clear correlation with time post transplantation. For example patient 5 sampled 12.5 month after transplantation showed higher IgA repertoire similarity compared to patient 1 sampled 1 month after transplantation (S4 Fig). Similarly, patients that underwent STD conditioning or RIC did not systematically differ with respect to repertoire similarity. For example, patient 8 exhibited higher similarity compared to the mean MHI value observed for RIC patients whereas the similarity is below average for patient 2. Moreover, it seems that the graft type does not determine the overall outcome, although we have only investigated few patients receiving bone marrow or CB grafts. Therefore, despite a great heterogeneity of repertoire similarity in individual patients, our data suggest that recipient B cells remain in the bone marrow after transplantation. However, persistence of recipient B cells in the bone marrow might reflect a complex combination of multiple factors, including the individual clinical course. Thus, future studies will need to consider the interplay of various parameters and potentially large cohorts of patients to identify the factors predicting Ig repertoire recovery.

### Post-transplantation repertoires show low diversity but retain highly mutated sequences

**Fig 3:**
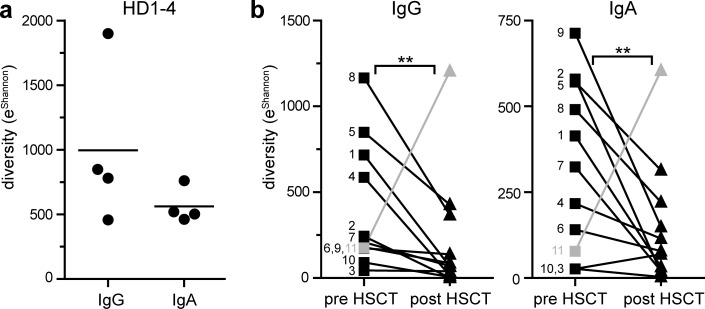
Repertoire diversity is reduced after allogeneic HSCT. (a) The Exponent Shannon (e^Shannon^) indices were calculated based on 4000 clustered IgA and IgG repertoires of four healthy donors (HD1-4). Symbols indicate individual samples, horizontal lines indicate the mean. (b) Diversity (e^Shannon^) of IgG and IgA repertoires pre- and post-HSCT was compared for eleven AML-patients. Exponent Shannon indices were calculated for each sample based on 2000 (patient 9) or 4000 (all other patients) clustered IgG and IgA sequences. Matched samples (pre- and post-transplantation) are connected by lines, numbers indicate the different patients. Statistical analysis was performed using paired T-test with Wilcoxon signed rank test, ** p< 0.01. Patient 11 (grey lines and symbols) was not considered for statistical analysis.

Post-transplantation repertoires appeared to be more narrow compared to pre-transplantation repertoires, i.e. post-transplantation repertoires comprised less unique CDR3 and more highly expanded sequences dominating the overall IgG (Fig 2B; S3 and S5A Figs) as well as IgA repertoires (S4 and S5B Figs). To quantify this observation, we calculated the Shannon index as a measure of Ig repertoire diversity. This index considers the number of unique clones and their abundance. Analyzing the Shannon index as function of input sequences, we observed that a total of 4000 input sequences were sufficient to describe 80% of the maximum diversity obtained for a doubling of input sequences (S5C Fig). Thus, in the following, we calculated diversity of Ig repertoires based on 4000 sequences per sample. In healthy bone marrow donors, IgG repertoire diversity tended to be higher compared to IgA diversity (Fig 3A). HSCT-patients showed great differences in pre-transplantation repertoire diversity, most likely reflecting individual disease course, but in general we noticed significant reduction of IgG and IgA repertoire diversity after transplantation (Fig 3B). There was no clear correlation between time post transplantation and Ig repertoire diversity. With one exception, reduced post-transplantation repertoire diversity was apparent in all samples irrespective of conditioning regimen, stem cell source, T cell reconstitution and immunosuppressive therapy. Notably, patient 7 underwent B cell depleting therapy with Rituximab in addition to cyclophosphamide and steroids for the treatment of post-transplant lymphoproliferative disease, and patient 4 was treated with high-dose steroids for acute GvHD °III, which both affect the B cell compartment. Indeed, diversity in these patients was low but did go below the overall diversity range detected in patients that did not undergo B cell affecting therapy.

**Fig 4:**
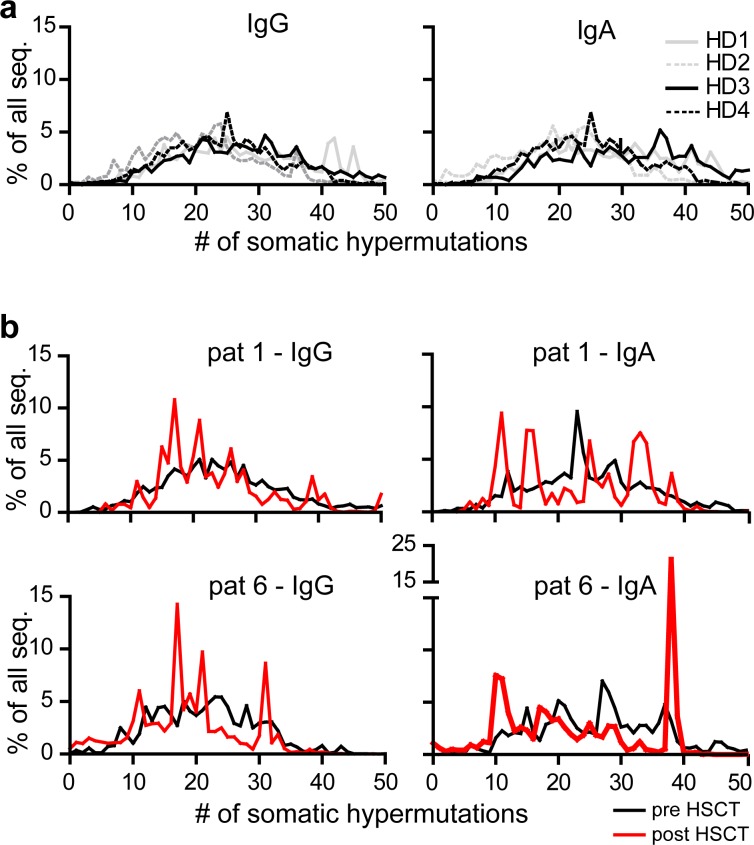
Switch Ig repertoires after HSCT comprise a spectrum of low and highly mutated clones. Numbers of somatic hypermutations in bone marrow switch IgA and IgG repertoires. Lines represent individual samples. Evaluation was based on all available sequences. Diagrams in (a) show results of four healthy donor repertoires (HD1-4), compared to (b) patient 1 and 6 evaluated separately for IgG (left column) and IgA (right column).

Low diversity of the post-transplantation repertoire might be reflective of a more immature state of the Ig repertoire. To explore this idea, we analyzed somatic mutation frequencies, which reflect affinity maturation and maturation/diversification of the Ig repertoire. In bone marrow of healthy donors the numbers of somatic mutations showed a broad distribution around a mean of about 25 (Fig 4A). Mutations were concentrated in CDR1 and CDR2 compared to framework regions 2 and 3 and replacement mutations exceeded silent mutations (S6A Fig), indicating antigen-driven affinity maturation. Consistent with more variable diversities of Ig repertoires in HSCT-patients somatic mutation frequencies were less evenly distributed (Fig 4B). Post-HSCT repertoires show a spiked pattern with peaks combining sequences showing the same number of somatic mutations. However, such peaks do not represent only one clonotype but are dominated in most cases by few clonotypes. This was also true for the two MGUS patients (patients 6 and 7). Yet, average numbers of somatic mutations were only slightly reduced in post- compared to pre-transplantation repertoires (S6B Fig). Thus, in HSCT-patients, highly mutated Ig sequences are present despite immunosuppression and low T cell counts in the peripheral blood early after transplantation as seen for patients 1, 2, 3, 4, 8, 9 and 10, which have still been under immunosuppressive therapy for GvHD-prophylaxis at the date of post-transplantation sampling (Table 1). Notably, average mutation frequencies were similar in post-transplantation samples compared to healthy donors, and mutation numbers did not correlate with time after transplantation (Fig 4 and S6 Fig). This raises the hypothesis that post-transplantation sequences might arise from pre-existing mutated B cell clones. These B cells might either derive from recipient cells that escape depletion during the conditioning phase or originate from affinity matured donor B cells. To explore this idea further, we compared the Ig repertoire of patient 9 pre- and post-transplantation to the Ig repertoire of the graft. B cell clones dominating the repertoire in the donor bone marrow showed only low similarity compared to the post-transplantation Ig repertoire. We therefore assume that mutated B cell clones dominating the Ig repertoire in the donor do not necessarily dominate the repertoire of mutated sequences in HSCT-patients.

Collectively, our data reveal key properties of the switch Ig repertoire in HSCT-patients. Based on the analysis of a small cohort of patients, we suggest that Ig repertoires in post-transplantation bone marrow showed low diversity despite the presence of highly mutated sequences in both STD and RIC regimens and irrespective of the graft type.

## Discussion

Multiple factors, such as conditioning regimen, graft source or immunosuppressive therapy, might affect the establishment of the B cell compartment after HSCT. A particularly powerful approach to study the development of the B cell system is Ig sequencing. Traditionally, these attempts relied on Sanger sequencing of comparably few clones and failed to comprehensively describe the complex B cell population. Here we used NGS to characterize the switch Ig repertoire in the bone marrow of HSCT-patients. This approach allows describing the development of B cells clones, the process of affinity maturation and diversity of the B cell repertoire [13]. Since we did not sort defined B cell populations, Ig repertoires analyzed herein comprise transcripts derived from B cells at various differentiation stages. Switch Ig repertoires include memory B cells and plasma cells but exclude B cell precursors and naïve B cells that have not yet switched to non IgM phenotypes. Still, the switch Ig repertoire is likely to be dominated by plasma cells that on average display higher Ig transcript levels when compared to other B cell populations.

Sequence reads corresponding to clonally related B cells present in healthy donors’ bone marrow greatly varied in their abundance. Whereas some sequences were highly abundant, other clones seemed represented by only very few sequences. This finding is reminiscent of our previous observation that IgA repertoires in murine gut comprised expanded as well as non-expanded B cell clones [15, 16] as well as Ig repertoire sequencing of human blood samples [17]. We observed similar characteristics for sorted murine gut plasma cells and human gut biopsies. Since samples in both studies almost exclusively contain plasma cells, expanded sequence clusters seems to be a distinguishing feature of the bone marrow compartment and reflect true differences in clonal sizes. However, a correlation of sequence reads and clonal sizes needs to be interpreted with great care. Different B cell populations differ with respect to transcript levels and our RNA-based approach does formally not allow quantifying cell numbers.

The human heavy chain B cell receptor repertoire has been estimated to contain between 0.5 x 10^6^ and 5 x 10^6^ clones [22, 23] and B cell repertoire sequencing in humans crucially needs to consider sample origin and size. Samples used in this study univocally are limited to a fraction of the overall bone marrow B cell compartment. Considering that in heathy donors plasma cells constitute about 1% of the leucocyte population [19], Ig repertoires obtained in this study seem unlikely to be limited by the amount of donor material. Indeed, clonally related sequences were readily identified in technical replicates and between Ig isotype repertoires obtained from the same donors but not between individuals. This indicates that comparably small bone marrow samples are sufficient to obtain relevant information about the composition of the bone marrow B cell compartment. This observation is surprising on the first sight, but it can be explained by the presence of expanded B cell clones. Expanded clones are likely to be sampled even in comparably small tissue samples and can be detected without the need to obtain fully saturated sequencing data. On the contrary, unexpanded clones and sample size limit formal repertoire similarity even in biological replicates to MHI similarity values well below 1.

Similar to previous studies analyzing the B cell repertoire in blood [24, 25], we found that bone marrow switch Ig repertoires appeared highly unique and characteristic of a given individual. This finding allowed us to separate donor and recipient derived B cell clones present in the post-transplantation repertoire of HSCT-patients and to identify recipient derived clonotypes that persisted in the bone marrow after HSCT. In fact, switch Ig repertoire analysis revealed varying but in some cases enormous persistence of recipient cells post-transplantation. This indicates that the recipient’s pre-transplantation state, i.e. the presence of vaccination titers, isoagglutinin titers and others, might not at all be entirely reset by HSCT. This finding is consistent with previous work describing persisting antibody titers [26, 27] in HSCT-patients.

Notably, persistence of recipient Ig-switched B cells was not evident from overall donor chimerism, determined in peripheral blood by PCR on short tandem repeats, which showed virtually complete reconstitution by donor cells. On average, 2.12±1.86% (mean±SD) of IgG and 4.36±3.62% (mean±SD) of IgA clonotypes detected before transplantation persisted after transplantation. Nearly half of them expanded post HSCT (1.01±1.23% IgG and 1.89±1.96% IgA clonotypes [mean±SD]), which demonstrates that these cells are not senescent but survived the conditioning regiment and proliferate to fill up the empty BM B cell niche.

Overall repertoire characteristics in post-transplantation samples were similar in RIC compared to STD patients as well as in recipients receiving bone marrow, PBSC or CB. Generally repertoires after transplantation were less diverse compared to healthy donors but showed surprisingly high numbers of somatic mutations. However, a total, maybe heterogeneous depletion of B cells after transplantation might have also contributed to decreased diversity. Previous efforts to study reconstitution of the B cell compartment in blood by CDR3 spectratyping did not identify strong diversity determining factors but indicated a slightly delayed reconstitution after RIC [28]. No such effect was evident in our data based on NGS analysis of bone marrow samples. This suggests that the conditioning therapy might not be a dominant determinant of post-transplantation B cell repertoire recovery.

Reduced B cell repertoire diversity in post-transplantation samples is in line with the MGUS-pattern frequently observed in serum protein electrophoresis of HSCT-patients. MGUS is considered to be a marker of B cell dysfunction after transplantation. It has been shown that MGUS after HSCT is associated with poor EBV-control and post-transplant lymphoproliferative disease (PTLD) [29, 30], which was also observed in one MGUS patient of our study cohort (patient 7). The other MGUS patient investigated (patient 6) suffered from GvHD, which has been associated with the presence of MGUS likewise [31]. Previous studies demonstrated that GvHD and/or immunosuppression can promote development of dominant B cell clones [32, 33]. Oligoclonal expansion of peripheral blood mononuclear cell repertoires from MGUS patients in non-HSCT context was recently analyzed in more detail [34]. We also observe highly expanded clones in the two MGUS patients included in our cohort, but the drop in diversity post-HSCT was comparable to non-MGUS patients. However, we cannot exclude that we did overlook the clones that manifest as MGUS because we limited our analysis to the VH1 family.

Unexpectedly, somatic mutation frequencies in donor-derived clones were comparable to the pre-transplantation situation as well as to mutation frequencies in persisting recipient clones. Notably, mutation frequencies did not very much increase with time post transplantation. We therefore suggest that the post-HSCT Ig repertoire is dominated by affinity matured B cell clones. It is unlikely that highly mutated B cells arise from naïve B cells within such comparable short period of time and in the presence of immunosuppression and low frequencies of T cells. Thus, highly mutated clones might either derive from the donor or represent recipient cells that had been in the repertoire before HSCT. In either case, it appears that the processes that drive the development of the B cell compartment during ontogeny fundamentally differ from the situation in AML patients after HSCT. These results are in line with earlier studies based on Ig segment usage analysis and CDR3 spectratyping (reviewed in [35, 36]).

Presence of affinity matured antibodies in post-HSCT samples raises the question whether patients might benefit from protective immunity elicited pre-HSCT. Our data demonstrate feasibility of Ig repertoire sequencing to describe the bone marrow B cell compartment and reveal a striking heterogeneity among patients. Since this study is a technical approach with a limited number of patients, our dataset is not sufficient to draw firm conclusions and therefore follow up studies as performed for T cell repertoires [37–39] are necessary to further evaluate B cell recovery after transplantation on clonal levels.

## Materials and Methods

### Patients

Eleven patients with AML were treated at the Department of Hematology, Hemostasis, Oncology and Stem-Cell Transplantation at Hannover Medical School between May 2000 and June 2014. Patients’ characteristics and disease status at transplantation are summarized in Table 1 and supplemental Table 1. Four healthy bone marrow donors served as controls.

### Immunoglobulin 454 sequencing

Human Ig repertoire information was generated from frozen total RNA isolated from mononuclear cells of either bone-marrow aspirates of AML-patients or bone-marrow donates of healthy donors. 0.5 μg RNA was used for cDNA synthesis using Superscript III (Invitrogen) with random hexamer primers according to manufacturer’s instructions. To generate sequence libraries of rearranged Ig, PCRs were performed using specific forward primers binding at the constant region of human IgA, IgG or IgE combined with a reverse primer specific for the human V_H1_ segment. Primers encode 4-nt multiple identifier sequences (MID), plus adaptor sequences (italic) required for pyrosequencing. The following primers were used for DNA-amplicon generation: IgA: Cα + V_H1_ (Cα 5’- *CGTATCGCCTCCCTCGCGCCATCAG*MIDGAATTCGAGTGGCTCCTGGGGGAAGA-3’; VH1 5’-*CTATGCGCCTTGCCAGCCCGCTCAG*GGCCTCAGTGAAGGTCTCCTGCAAG-3’); IgG: Cγ + V_H1_ (Cγ 5’-*CTATGCGCCTTGCCAGCCCGCTCA*MIDGTTCCACGACACCGTCACC-3’; V_H1_ 5’- *CGTATCGCCTCCCTCGCGCCATCAG*GGCCTCAGTGAAGGTCTCCTGCAAG-3’); IgE: Cε + V_H1_ (Cε 5’-*CTATGCGCCTTGCCAGCCCGCTCAG*MIDAAGGGGAAGACGGATGGGCTCTG-3’; V_H1_ 5’- *CGTATCGCCTCCCTCGCGCCATCAG*GGCCTCAGTGAAGGTCTCCTGCAAG-3’). PCR conditions were as follows: 95°C, 4 min; 35 x (94°C, 30s; 62°C, 30s; 72°C, 35s) and 72°C 10 min. For some samples a semi-nested PCR approach was required, performing a pre-amplification PCR of 25 cycles using outer primer V_H1,5_ (5‘-*CGTATCGCCTCCCTCGCGCCATCAG*GAGGTGCAGCTGTGCAG-3’). PCR-products were purified by gel electrophoresis and quantified by Quant-iT dsDNA HS Assay kit (Invitrogen). Amplicons were prepared with the GS FLX Titanium SV emPCR kit (Lib-A) for 454 pyrosequencing on the Genome Sequencer FLX system (Roche) as described by the manufacturer.

### Sequence analysis

Ig sequence analysis was performed as previously described for murine IgA repertoires [16] with some complementation. In brief: Sequences were assigned to individual samples by MIDs and further analyzed with ImMunoGeneTics (IMGT) HighV-QUEST [20, 21]). All sequences were compared against the IMGT reference database, resulting in detailed information about FR and CDR sequences, VDJ-usage and mutation patterns compared to germline segments. Results obtained were further analyzed with in house VBA-scripts. Clonally related sequences were grouped as clonotypes, based on 95% CDR3 sequence identity and same VJ-usage, a process further referred to as clustering performed by pairwise sequence alignment using USEARCH 5.0.1 [40]. For evaluation 4000 sequences were taken into account, if not stated differently. Repertoire similarity was calculated on clustered sequences as Morisita-Horn similarity index (MHI): $MHI=\frac{2\sum ({an}_{i}∙{bn}_{i})}{(da+db)∙aN∙bN}\;da=\frac{\sum {an}_{i}^{2}}{{aN}^{2}}\;db=\frac{\sum {bn}_{i}^{2}}{{bN}^{2}}$, in which aN represents total number of clusters per given sample and an_j_ equals the number of clusters of the type i as part of aN. To visualize repertoire similarity, heatmaps of clustered sequences were generated showing the 100 most frequent clonotypes of the reference sample and their abundance within compared repertoires; frequencies are reflected by color code. For diversity estimation Shannon indices were calculated using R studio library “vegan” (version 0.94.110; http://www.r-project.org/; http://rstudio.org/). Shannon Index = —∑ p_i_ x ln p_i_; p_i_ = n_i_ / N. Operated on sequencing data, N reflects the number of all CDR3 sequences detected in the repertoire, n_i_ equals the number of a distinct CDR3 sequence i within the analyzed repertoire, resulting in p_i_ as ratio of CDR sequence i within the sequence set. Exponent Shannon was chosen to express repertoire diversity. Mutation frequencies were calculated as number of mutations divided by the number of all nucleotides in the region investigated.

### Statistical analysis

Statistical analysis was performed using Prism software (GraphPad Software). Two groups were compared by Wilcoxon signed rank test. For comparison of more than two groups Kruskal-Wallis test was performed. P-values are indicated following: *, p<0.05; **, p<0.01; ***, p<0.001. Some data are presented as mean+SD.

### Study approval

This study was done in accordance with the declaration of Helsinki and approved by the institutional review board (number 2594–2015). Written informed consent was received from participants prior to inclusion in the study.

## Supporting Information

S1 Fig Technical replicates show highly reproducible results. The IgG-repertoire of healthy donor 4 (replicate I) was re-investigated by independent PCR-amplification (replicate II) and independent cDNA synthesis plus PCR-amplification from the same starting material (replicate III). The plot illustrates individual clones (filled circles) present in both samples compared, based on evaluation of 2500 clustered sequences per replicate. Open circles indicate clonotypes of the same frequency, with blue numbers indicating if more than 2. Pearson correlation (r) was calculated including clones present in both replicates only (465 pairs left dot plot; 436 pairs right dot plot).
(TIF)

S2 Fig Ig-subclass composition of human bone marrow samples. (a) IgA, IgG and IgE repertoires of healthy donors 2–4 (HD2-4) were analyzed for CDR3-sequence overlap. Related clones were clustered and sorted according to the 100 most abundant clonotypes present in the IgA, IgG, or IgE repertoires and displayed as heatmap. CDR3 similarity in the IgA, IgG and IgE repertoires of HD2-4 is expressed as Morisita-Horn index (MHI). (b) IgG and IgA sequences of HD1-4 bone marrow samples were assigned to the different subclassesIgA1 and IgA2 as well as IgG1-4. Total numbers of sequences assigned to the different subclasses are listed in the table. (c) Heatmaps illustrate CDR3 overlap between Ig-subclasses of HD4. Related clones of each subclass repertoire were clustered (4000 sequences if available, otherwise sequence numbers according to the above table; 95% CDR3 sequence identity; same VJ-usage), and sorted according to the 100 most abundant clonotypes present. MHI-values of pairwise comparisons are listed in the table.
(TIF)

S3 Fig IgG repertoire dynamics of AML-patients treated by allogeneic HSCT. Complements Fig 2 with data of the remaining 9 patients listed in Table 1. Evaluation is based on 4000 clustered sequences each (only 2000 sequences for patient 9). Heatmaps illustrate shared clonotypes for the 100 most frequent clonotypes before and after HSCT. Pre- and post-HSCT repertoire similarity is quantified as Morisita-Horn index (MHI).
(TIF)

S4 Fig IgA repertoire dynamics of AML-patients treated by allogeneic HSCT. IgA sequences of eleven patients pre- and post-HSCT (patient 1 and patient 9: 2000 sequences; other patients: 4000 sequences) were assigned to clonotypes (see **Fig 1** for details). Heatmaps show CDR3-overlap of the 100 most abundant IgA clonotypes. Repertoire similarity is described as Morisita-Horn index (MHI).
(TIF)

S5 Fig Exponent Shannon as function of number of input sequences. (a, b) For each patient clonotypes were sorted by frequency and their abundance displayed IgG and IgA repertoires pre- (blue line, filled area) and post-HSCT (red line, non-filled area). Numbers indicate the amount of unique clones (x-axis) and number sequences assigned to each individual clonotype (y-axis). Graphs show the clonal composition of IgG (a) and IgA repertoires (b). The bar graph depicts the ratio of unique CDR3 sequences pre- and post-transplantation in individual patients; ratio above 1 indicates a reduced and a ratio below 1 an increased sample richness post-HSCT (c) Exponent Shannon, expressing sample diversity, was calculated for varying numbers of clustered input IgG sequences (95% CDR3 sequence identity; same VJ-usage) derived from healthy donor 3 (HD3) and patient 1 before transplantation (1_pre_ HSCT).
(TIF)

S6 Fig Overall mutation frequency is not impaired after transplantation. (a) IgA and IgG repertoires of four healthy donor (HD1-4) and eleven patient samples pre- as well as post-HSCT (pat 1–11) were analyzed for frequencies of silent and replacement mutations within frame work regions 2 and 3 (FR 2, 3) and complementarity determining regions 1 and 2 (CDR1, 2). Error bars depict mean+SD with n = 4 (HD1-4) or n = 11 (pat1-11). (b) Average number of mutations for each patient, listed separately for IgA and IgG repertoires pre- and post-HSCT. Matched samples are connected by lines, all sequences were taken into account. (c) Graphs depict the frequencies of IgG and IgA sequences containing the indicated number of somatic hypermutations (d) For patient 9 Ig repertories pre- and post- transplantation were compared to the respective donor-repertoire; evaluation is based on 1900 clustered sequences. Heatmaps show overlap of the 100 most frequent clonotypes, compared to their frequencies in the other repertoires. Morisita-Horn indices (MHI) were calculated to quantify overall repertoire similarity.
(TIF)

S1 Table Additional clinical information of AML-patients investigated for repertoire analysis before and after allogeneic HSCT. (DOCX)
