# Supplementary figures and images for: VH1 Family Immunoglobulin Repertoire Sequencing after Allogeneic Hematopoietic Stem Cell Transplantation
Source: PLoS One. 2017 Jan 17;12(1):e0168096. doi: 10.1371/journal.pone.0168096 (PMC5240918; doi:10.1371/journal.pone.0168096)

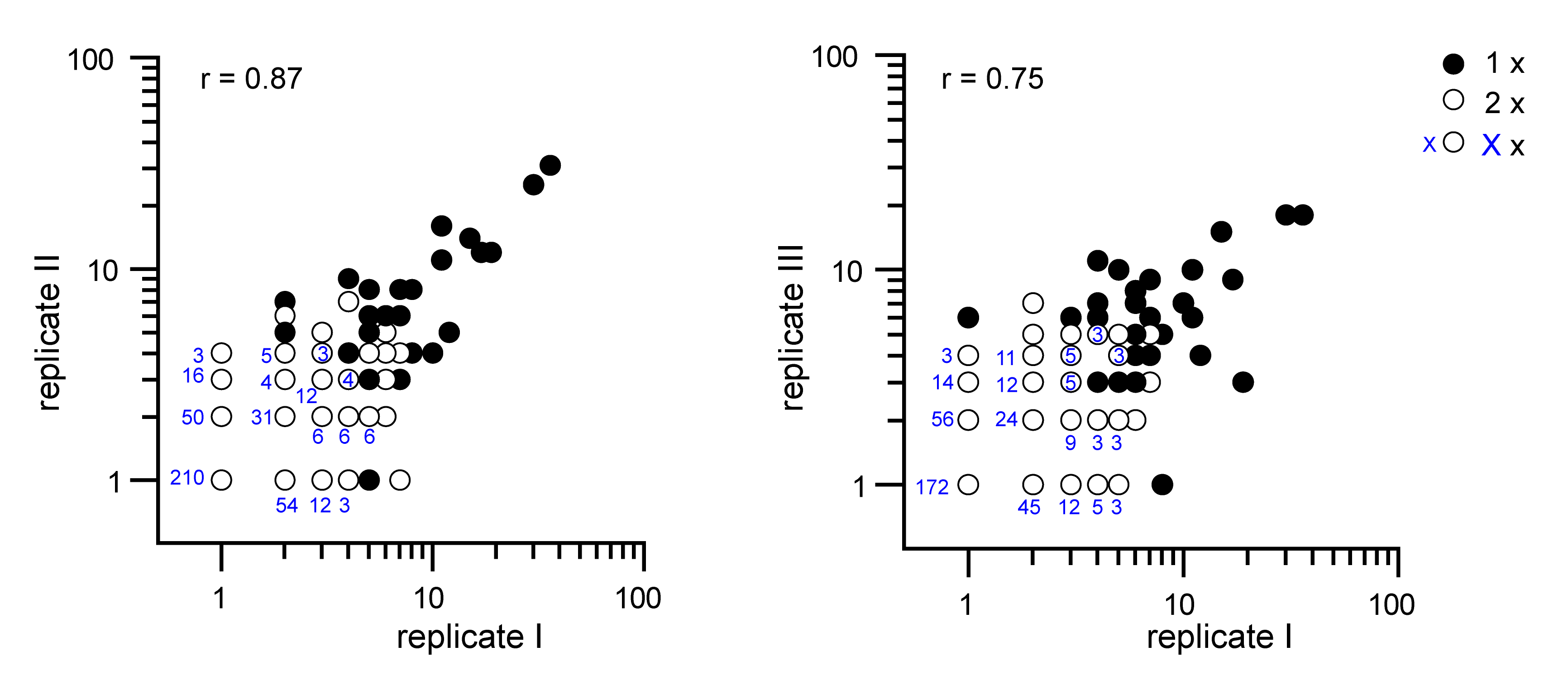

Supplement: S1 Fig — The IgG-repertoire of healthy donor 4 (replicate I) was re-investigated by independent PCR-amplification (replicate II) and independent cDNA synthesis plus PCR-amplification from the same starting material (replicate III). The plot illustrates individual clones (filled circles) present in both samples compared, based on evaluation of 2500 clustered sequences per replicate. Open circles indicate clonotypes of the same frequency, with blue numbers indicating if more than 2. Pearson correlation (r) was calculated including clones present in both replicates only (465 pairs left dot plot; 436 pairs right dot plot). (TIF) [file pone.0168096.s001.tif]

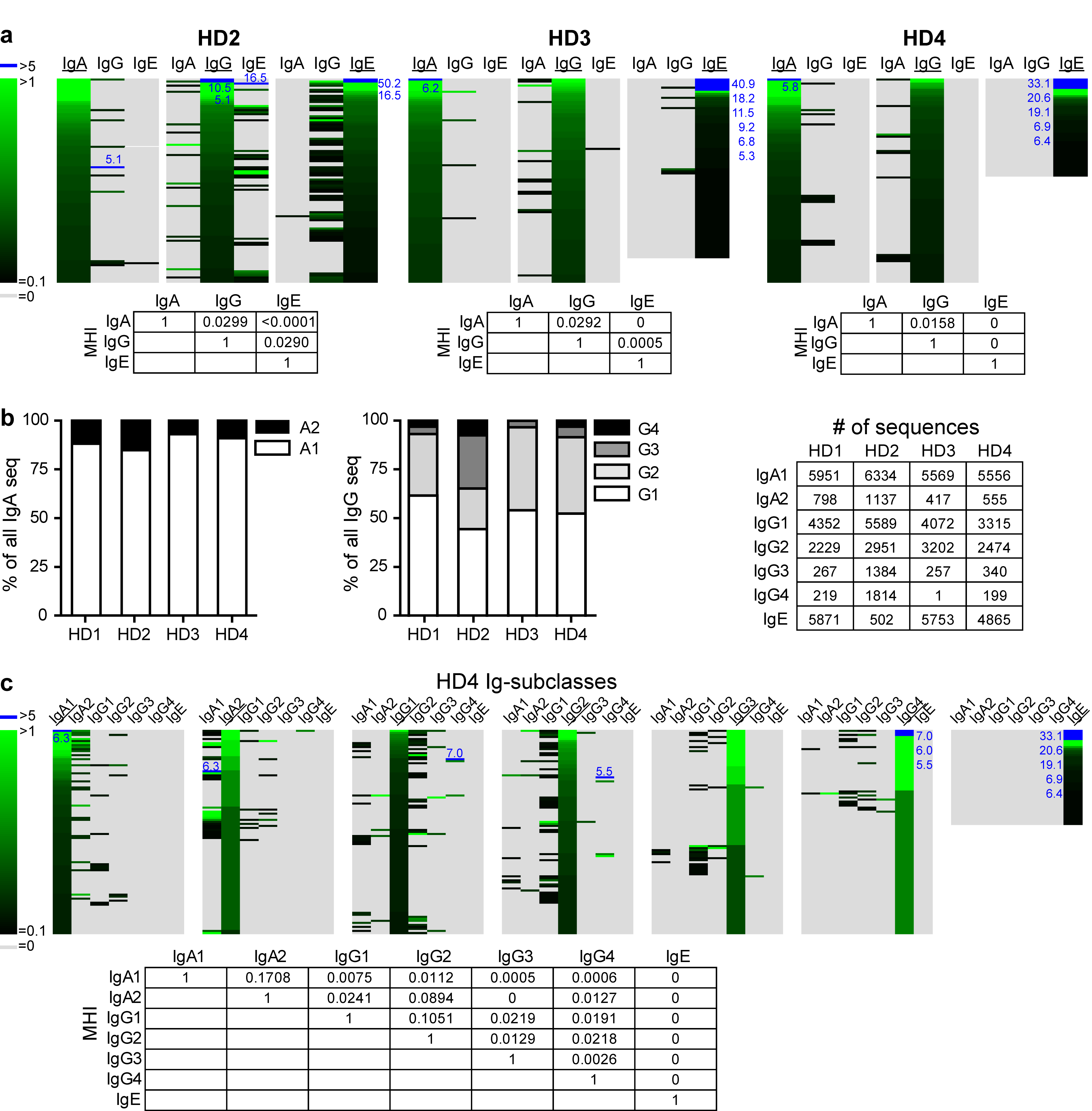

Supplement: S2 Fig — (a) IgA, IgG and IgE repertoires of healthy donors 2–4 (HD2-4) were analyzed for CDR3-sequence overlap. Related clones were clustered and sorted according to the 100 most abundant clonotypes present in the IgA, IgG, or IgE repertoires and displayed as heatmap. CDR3 similarity in the IgA, IgG and IgE repertoires of HD2-4 is expressed as Morisita-Horn index (MHI). (b) IgG and IgA sequences of HD1-4 bone marrow samples were assigned to the different subclassesIgA1 and IgA2 as well as IgG1-4. Total numbers of sequences assigned to the different subclasses are listed in the table. (c) Heatmaps illustrate CDR3 overlap between Ig-subclasses of HD4. Related clones of each subclass repertoire were clustered (4000 sequences if available, otherwise sequence numbers according to the above table; 95% CDR3 sequence identity; same VJ-usage), and sorted according to the 100 most abundant clonotypes present. MHI-values of pairwise comparisons are listed in the table. (TIF) [file pone.0168096.s002.tif]

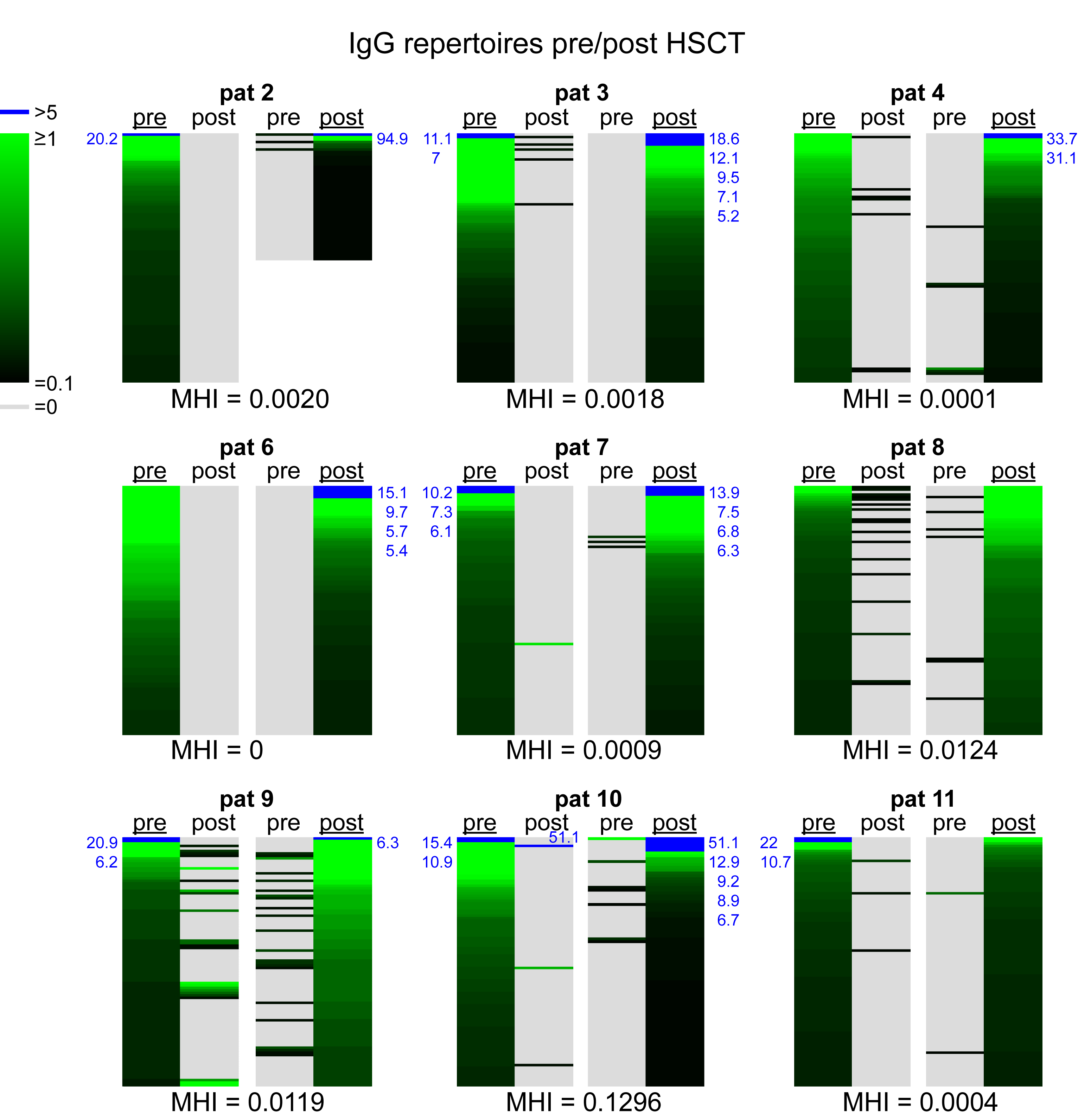

Supplement: S3 Fig — Complements Fig 2 with data of the remaining 9 patients listed in Table 1. Evaluation is based on 4000 clustered sequences each (only 2000 sequences for patient 9). Heatmaps illustrate shared clonotypes for the 100 most frequent clonotypes before and after HSCT. Pre- and post-HSCT repertoire similarity is quantified as Morisita-Horn index (MHI). (TIF) [file pone.0168096.s003.tif]

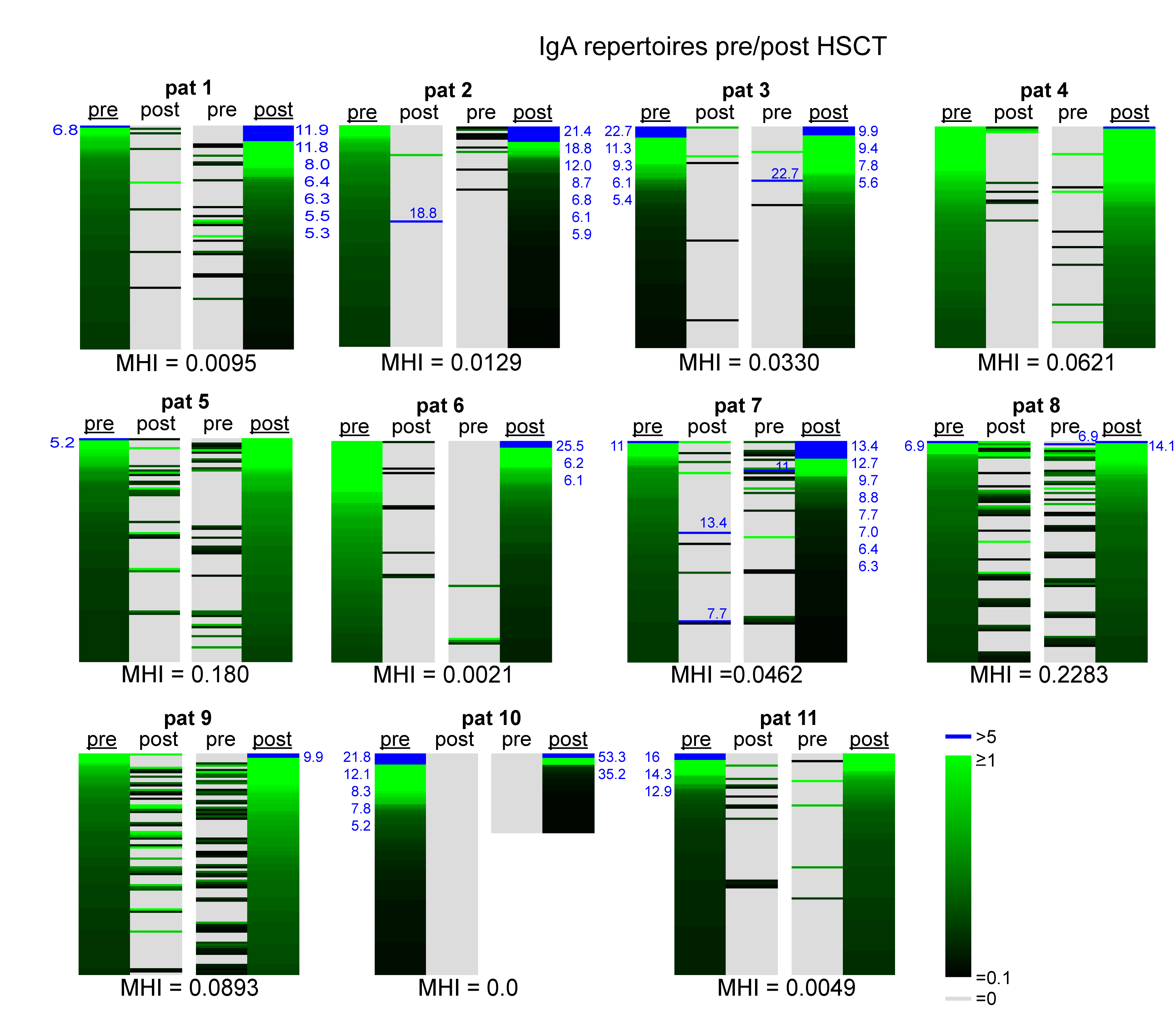

Supplement: S4 Fig — IgA sequences of eleven patients pre- and post-HSCT (patient 1 and patient 9: 2000 sequences; other patients: 4000 sequences) were assigned to clonotypes (see Fig 1 for details). Heatmaps show CDR3-overlap of the 100 most abundant IgA clonotypes. Repertoire similarity is described as Morisita-Horn index (MHI). (TIF) [file pone.0168096.s004.tif]

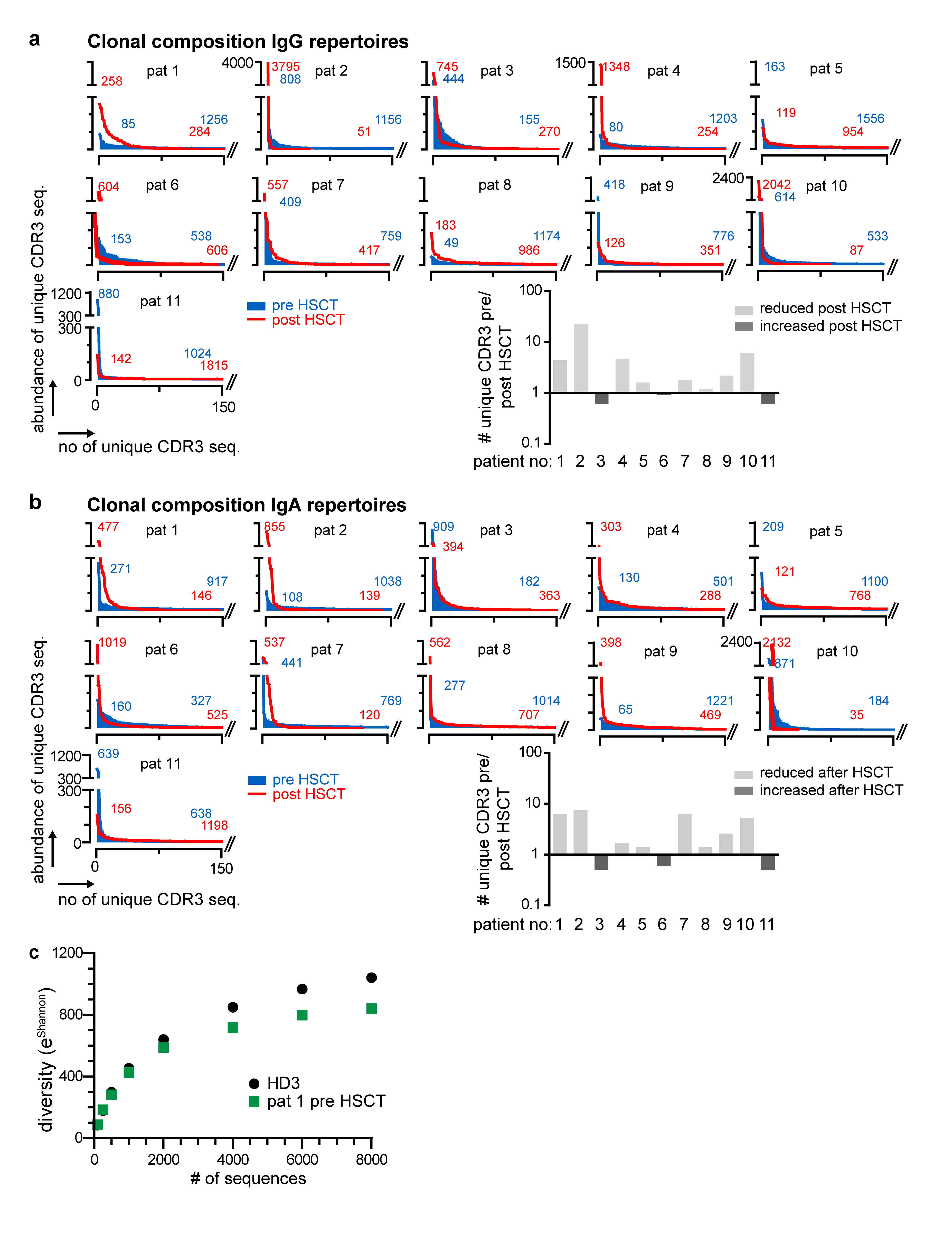

Supplement: S5 Fig — (a, b) For each patient clonotypes were sorted by frequency and their abundance displayed IgG and IgA repertoires pre- (blue line, filled area) and post-HSCT (red line, non-filled area). Numbers indicate the amount of unique clones (x-axis) and number sequences assigned to each individual clonotype (y-axis). Graphs show the clonal composition of IgG (a) and IgA repertoires (b). The bar graph depicts the ratio of unique CDR3 sequences pre- and post-transplantation in individual patients; ratio above 1 indicates a reduced and a ratio below 1 an increased sample richness post-HSCT (c) Exponent Shannon, expressing sample diversity, was calculated for varying numbers of clustered input IgG sequences (95% CDR3 sequence identity; same VJ-usage) derived from healthy donor 3 (HD3) and patient 1 before transplantation (1pre HSCT). (TIF) [file pone.0168096.s005.tif]

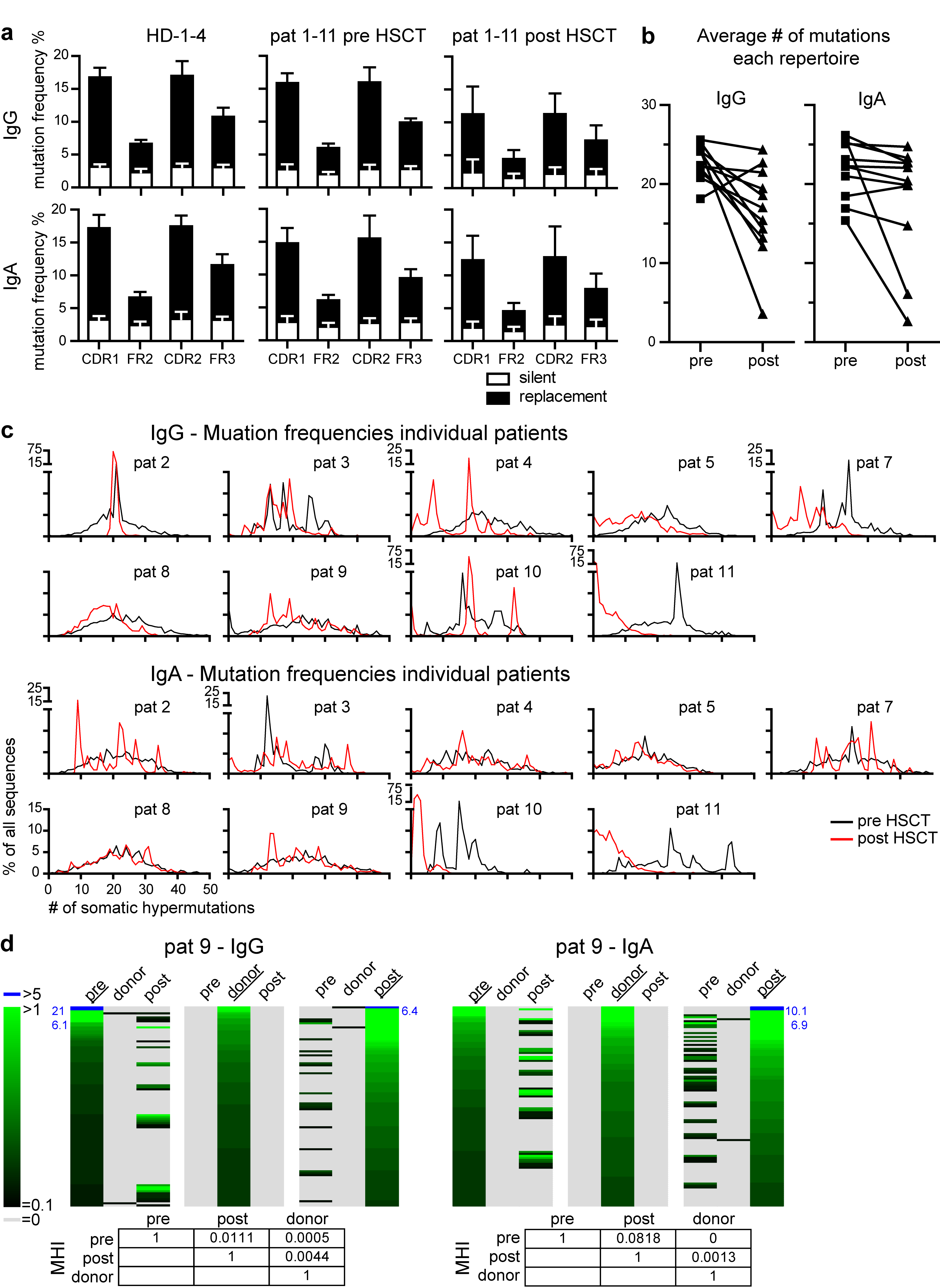

Supplement: S6 Fig — (a) IgA and IgG repertoires of four healthy donor (HD1-4) and eleven patient samples pre- as well as post-HSCT (pat 1–11) were analyzed for frequencies of silent and replacement mutations within frame work regions 2 and 3 (FR 2, 3) and complementarity determining regions 1 and 2 (CDR1, 2). Error bars depict mean+SD with n = 4 (HD1-4) or n = 11 (pat1-11). (b) Average number of mutations for each patient, listed separately for IgA and IgG repertoires pre- and post-HSCT. Matched samples are connected by lines, all sequences were taken into account. (c) Graphs depict the frequencies of IgG and IgA sequences containing the indicated number of somatic hypermutations (d) For patient 9 Ig repertories pre- and post- transplantation were compared to the respective donor-repertoire; evaluation is based on 1900 clustered sequences. Heatmaps show overlap of the 100 most frequent clonotypes, compared to their frequencies in the other repertoires. Morisita-Horn indices (MHI) were calculated to quantify overall repertoire similarity. (TIF) [file pone.0168096.s006.tif]
