# Supplementary material for: VH1 Family Immunoglobulin Repertoire Sequencing after Allogeneic Hematopoietic Stem Cell Transplantation
Source: PLoS One. 2017 Jan 17;12(1):e0168096. doi: 10.1371/journal.pone.0168096 (PMC5240918; doi:10.1371/journal.pone.0168096)
Supplement: S1 Table — (DOCX) [file pone.0168096.s007.docx]

Table S1. **Additional clinical information of AML-patients investigated for repertoire analysis before and after allogeneic HSCT.**

| **Patient number** | **Donor Age** | **Donor Sex** | **GvHD-prophylaxis** | **Discontinuation of GvHD-prophylaxis** (days after Tx) | **Donorcell-chimerism** (%) | | **Plasmacell Freq.^§^** (%; norm. 0,2-2,2) | | **Ig levels** (g/L) | | | **CD4^+^ T cells/µl** (norm.: 500-1200) |
| --- | --- | --- | --- | --- | --- | --- | --- | --- | --- | --- | --- | --- |
|  |  |  |  |  | BM | PB | before | after | G (norm.: 7-16) | A (norm.: 0,7-4) | M (norm.: 0,4-2,3) |  |
| 1 | 47 | m | ATG/CSA/MMF | 100 | 100 | 100 | 0.25 | n.a. | 6.56 | 1.25 | 0.27 | 22 |
| 2 | 34 | m | ATG/CSA/MTX | 100 | 100 | 100 | 0.25 | 0.00 | 6.03 | 0.44 | 0.43 | 29 |
| 3 | 29 | m | ATG/CSA/MTX | 136 | 100 | 100 | 3.75 | 1.25 | 7.01 | 1.54 | 0.53 | 221 |
| 4 | 41 | m | ATG/CSA/MMF | 345 | 100 | 100 | 5.75 | 1.25 | 11.3 | 0.99 | 1.8 | 304 |
| 5 | 22 | f | ATG/CSA/MTX | 100 | 85 | 18 | 0.00 | 0.00 | 13.7 | 1.56 | 2.93 | n.a. |
| 6 | 28 | f | ATG/CSA/MMF | 104 | 100 | 100 | 10.50 | 1.00 | 10 | 0.79 | 0.39 | 180 |
| 7 | 26 | m | ATG/CSA/MMF | 130 | 86 | 57 | 0.25 | 0.50 | 13.7 | 0.54 | 0.91 | 296 |
| 8 | 39 | f | CSA/MTX | 100 | n.a. | n.a. | n.a. | n.a. | 3.17 | 0.23 | 0.17 | 93 |
| 9 | 46 | m | ATG/CSA/MTX | 100 | 100 | 100 | 0.25 | 0.00 | 25.5 | 0.62 | 0.45 | n.a. |
| 10 | 0 | m/n.a. | ATG/CSA/MMF | 130 | 100 | 100 | 0.00 | 0.25 | 6.33 | 0.41 | 0.56 | 225 |
| 11 | 0 | f/male | CSA/MMF | 125 | 100 | 100 | 7.75 | 0.50 | 10.1 | 1 | 1.36 | 105 |

^§^ (400 BM cells counted)
